# Burnout and intentions to quit the practice among community pediatricians: associations with specific professional activities

**DOI:** 10.1186/s13584-018-0268-2

**Published:** 2019-01-04

**Authors:** Zachi Grossman, Gabriel Chodick, Talma Kushnir, Herman Avner Cohen, Gil Chapnick, Shai Ashkenazi

**Affiliations:** 1 Pediatric clinic, Maccabi Healthcare Services, 26 Rofe Hamachtarot, 69372 Tel Aviv, Israel; 2 Maccabitech, Maccabi Healthcare Services, Tel-Aviv, Israel; 3 Sackler Faculty of Medicine, Tel-Aviv University, Tel-Aviv, Israel; 4 Department of Psychology, Faculty of Social Sciences, Ariel University, Ariel, Israel; 5 Pediatric Community Clinic, Clalit Healthcare Services, Petah Tikva, Israel; 6 Maccabi Healthcare Services, Kfar Saba, Israel; 7 Schneider Children’s Hospital, Petah Tikva, Israel; 8 Adelson School of Medicine, Ariel University, Ariel, Israel

**Keywords:** Burnout, Pediatricians, Satisfaction, Tutoring, Research

## Abstract

**Background:**

Burnout is an occupational disease expressed by loss of mental and physical energy due to prolonged and unsuccessful coping with stressors at work. A prior survey among Israeli pediatricians published in 2006 found a correlation between burnout and job structure match, defined as the match between engagement with, and satisfaction from, specific professional activities. The aims of the present study were to characterize the current levels of burnout and its correlates among community pediatricians, to identify changes over time since the prior survey, and to identify professional activities that may reduce burnout.

**Methods:**

A questionnaire was distributed among pediatricians both at a medical conference and by a web-based survey.

**Results:**

Of the 518 pediatricians approached, 238 (46%) responded to the questionnaire. High burnout levels were identified in 33% (95% CI:27–39%) of the respondents. Higher burnout prevalence was found among pediatricians who were not board-certified, salaried, younger, and working long hours. The greater the discrepancy between the engagement of the pediatrician and the satisfaction felt in the measured professional activities, the greater was the burnout level (*p* < 0.01). The following activities were especially associated with burnout: administrative work (frequent engagement, disliked duty) and research and teaching (infrequent engagement, satisfying activities).

A comparison of the engagement-satisfaction match between 2006 and 2017 showed that the discrepancy had increased significantly in research (*p* < 0.001), student tutoring (*P* < 0.001), continuing medical education and participation in professional conferences (*P* = 0.0074), management (*p* = 0.043) and community health promotion (*P* = 0.006). A significant correlation was found between burnout and thoughts of quitting pediatrics or medicine (*p* < 0.001).

**Conclusions:**

Healthcare managers should encourage diversification of the pediatrician’s job by enabling greater engagement in the identified “anti-burnout” professional activities, such as: participation in professional consultations, management, tutoring students and conducting research.

## Background

Physician burnout is an occupation-related syndrome involving emotional exhaustion, depersonalization, and a sense of reduced personal accomplishment [1–3]. Studies indicate that physician burnout influences quality of care, patient safety, and patient satisfaction [4–6]. Physician burnout and distress have also been linked to physician’s prescribing habits, test ordering, risk of malpractice suits, and whether or not patients adhere with the physicians’ medical recommendations [7].

Burnout has reached epidemic levels, as documented in national studies of both practicing and in training and practicing physicians [1]. Many of the specialties at the front line of access to care (e.g., family medicine, general internal medicine, and emergency medicine) are at highest risk of burnout [7]. Reported rates of physician burnout have ranged from 30 to 65% across medical specialties, with general pediatricians (∼35%) falling among specialties with the lowest rates and subspecialty pediatrics (∼40%) in the lowest third [8].

A previous study published in 2006 among Israel pediatricians showed that work structure match was one of the correlates of burnout. Specifically, burnout was associated with the existence of a substantial discrepancy between the level of engagement demanded by the job, and the extent to which pediatricians find satisfaction in what they are doing [9]. Burnout was associated with infrequent performance of satisfying activities (e.g. research, tutoring medical students), and frequent engagement in disliked duties (e.g. administrative activities) [9].

The aims of the present study were to evaluate Israeli pediatricians’ burnout in 2017, to compare job structure and other burnout correlates between 2006 and 2017, and to highlight interventions that can reduce the burnout.

## Methods

### Study sample

Structured validated questionnaires were distributed to pediatricians in two sessions: to Israel Pediatric Research in Office setting NETwork (IPRONET) members and to Israel Ambulatory Pediatric Association conference attendees.

#### IPRONET members

The Israel Pediatric Research in Office Setting (IPROS) was established in 1995 as a network of Israeli pediatricians who are willing to do research in their clinics [10]. The IPROS Network mailing list, IPRONET, is an electronic mailing list that can be joined voluntarily by all pediatricians in Israel. Initially it was established as an interest group intended to promote research, but as the years went by, it became more like an active forum of pediatricians. As such, it is now more like an open forum, a place where research proposals are still being discussed, but other issues relating to child health in Israel - policy controversies, clinical dilemmas and also information on upcoming conferences – are more dominant. Once a year a call is sent to all 2700 Israeli pediatricians [11] to join IPRONET.

An electronic version of the questionnaire was posted online, and a link was sent to pediatricians who are members of the list.

#### Conference attendees

Questionnaires were handed out to pediatricians attending the Israel Ambulatory Pediatric Association annual conference held in July 2017. The call to attend the Ambulatory Association conferences is sent to all 2700 Israeli pediatricians by organizational emails, and is also posted in IPRONET.

Since most pediatricians in the country work also at the ambulatory sector, these conferences are highly attractive. All attendees were employees of the four Health Maintenance Organizations (HMOs) in Israel. Forty one percent, 20, 28 and 11% were employed by Clalit, Maccabi, Meuhedet and Leumit organizations respectively.

Questionnaires were distributed in the conference only to non-IPRONET member pediatricians, to avoid overlapping results with the previous group.

#### Representativeness and comparability

The general characteristics of the study participants (age, gender, country of medical studies) were compared to those of Israeli pediatricians which were published in Israel’s Ministry of Health (IMOH) report on health professionals in 2015 [11]. Currently this is the only source of characteristics of pediatric workforce that is available.

In addition, the results of the conference attendees group and the results of the IPRONET members group were analyzed separately and then compared to determine the homogeneity of the sample.

### Research instruments

#### Socio-demographic details

Sex, age, number of children, country of birth, country of medical school graduation, professional status (board certification in pediatrics), academic position, managerial status (director of clinic: yes/no), years in practice, working place (community clinic, child health center, hospital, combination), hours in clinic per week, type of contract (independent, salaried, combination), ride time to work.

#### The job structure match measure

This measure has been developed for a research on occupational stress and burnout among primary care physicians [12]. It has also been used previously in samples of Israeli community pediatricians [9] and occupational physicians [13]. It combines two aspects of the job: perceived workload and satisfaction from nine specific professional tasks.

This measure is based on the premise that frustration, distress and burnout are associated with the existence of a substantial discrepancy between the level of engagement demanded by the job, and the extent to which pediatricians find satisfaction in what they are doing. This hypothesis is based on congruence models in psychology and occupational health. According to such models, individuals with certain needs are most likely to seek environments that are congruent with their perceived needs. Dissonance, or mismatch, between their preferred needs and actual activities could result in frustration. When the individual must function in a dissonant milieu, stress and discomfort follow. A prominent health version of this approach is the Person-Environment-Fit model (P-E Fit) which postulates that the discrepancy between what a person (P) wants and what he/she gets from work (E) creates job distress and dissatisfaction [14].

##### Perceived workload

The extent of engagement in each of the following nine professional activities: treating acute illnesses in regular clinic visits; family interventions initiated by the pediatrician; consulting parents about vaccinations, child development and growth for those children that failed initial screening; managerial tasks (such as defining priorities in the clinic, integration of staff functions); community health promotion activities; administrative duties including paperwork and telephone calls related to patients; professional consultations and participation in continuing medical education (CME) activities; research; and tutoring residents and medical students. The response scale for all items ranged from 1(“Not at all”) to 4 (“A lot”).

##### Task satisfaction

Each of the above activities was also rated on the amount of satisfaction it provided, ranging from 1 (“Not at all”) to 4 (“A lot”). The sum of the scores in each of the scales divided by the number of items (9) represents the average level of workload and satisfaction from these activities.

##### Computation of workload-satisfaction match

In order to assess the level of matching between perceived workload and satisfaction, a match score was computed for each of the activities, as shown below in the data analysis section.

#### Burnout

Burnout was evaluated by using the Maslach Burnout Inventory (MBI), most widely used by researchers for measuring burnout among healthcare professionals [15]. The MBI is a 22-item self-reported questionnaire, which is well recognized and widely used to measure burnout in relation to occupational stress. It has three subscales: personal accomplishment (measured by 8 items), depersonalization (DEP) (measured by 5 items) and emotional exhaustion (EE) (measured by 9 items). Responses are rated for each item according to the frequency on a 7-point scale from ‘almost never’ to ‘every day’.

The reliability scores were: for the questionnaire – 0.86, for EE burnout– 0.83, for DEP burnout– 0.74.

#### Quitting the practice

Thoughts of quitting the pediatric practice and of quitting medicine in general were evaluated using two Likert scales ranging from 1 (“Never”) to 5 (“Often”).

### Data analysis

#### Computation of workload-satisfaction match

In order to assess the level of matching between perceived workload and satisfaction, a match score ranging between *−* 1 and + 1 was computed as follows. For each activity of the individual respondent, a score of ‘1’ was assigned only when both the levels of engagement and satisfaction were high (3 or 4). A score of ‘*−* 1’ was assigned to all items in which one of the variables (either engagement level or satisfaction) was high (3 or 4) or low (1 or 2), and the other variable was the opposite. In all other cases, a score of ‘0’ was assigned. The final match score was the sum of these item scores divided by 9 (the number of activities). A high score denotes high matching between workload and satisfaction e.g. intense engagement and high satisfaction; and a low score reflects high discrepancy i.e. either high workload and low satisfaction, or low workload and high satisfaction.

Workload and task satisfaction were studied in two ways: 1. as scores related to each of the nine specific professional tasks (e.g. tutoring residents and medical students); 2. as aggregate scores, i.e. the mean scores of nine workload and nine satisfaction items.

#### Burnout

In accord with other studies on physician burnout we referred only to the EE and DEP indices to assess two separate measures of burnout. First, burnout level was assessed as a global score (averaging all EE and DEP items). Second, burnout frequency (yes/no) was defined as having either an EE score of > = 27, or a DEP score of > = 10.

Differences in overall burnout score and in each of the two Maslach Burnout Inventory sub-scales (EE and DEP), as well as aggregate scores of workloads, satisfaction, match scores, and socio-demographic details, were assessed using t-tests or analysis of variance for categorical variables and Pearson correlation coefficients for continuous variables. Correlation coefficients lower than 0.30 were considered weak, those of 0.30–0.50 moderate, and those higher than 0.50 were considered strong. For all performed analyses of variance partial eta-squared effect sizes were calculated, indicating the explained proportion variation caused by group differences. Values of 0.01, 0.06 and 0.14 reflect small, medium and large effects, respectively. Finally, a series of multiple linear regression analyses were conducted. All analyses were performed using IBM-SPSS Statistics for Windows, Version 22.0(Armonk, NY: IBM Corp). General linear modeling analyses were used to assess the relationship between burnout and thoughts of quitting either the pediatric practice or medicine. Comparison of match scores with respective scores published in 2006 was performed using t-tests.

## Results

Two hundred and thirty-eight pediatricians responded to the survey, response rate (RR) = 46%. In the conference, 118 responses were collected from 220 non IPRONET members (RR = 53%), and there were 120 responses in the IPRONET members group (RR = 40%). The higher response in the conference may be attributed probably to the face to face recruitment.

### Representativeness and comparability

The representativeness of the responding physicians was evaluated by comparing characteristics of the study participants with those of Israeli pediatricians [11], regarding age groups distribution (53% under 55 years in study participants vs. 51% in the IMOH report), gender (55% vs. 52% females) and country of medical studies (Israel, 65% vs. 59%). Separate analyses of both the conference attendees’ group and the IPRONET group and comparison between the two groups is presented in Table 1. Overall, with the exception of Burnout DEP, results were quite comparable between the two study groups. Considering the fact that a high Burnout DEP score is defined as a score above 10, the difference between the groups, though significant, apparently has little if any importance.

**Table 1:** Comparison between groups

		IPRONET members		Meeting attendees		p
		Mean	SD	Mean	SD	
SEX	M n, %	56	47.9%	51	42.5%	0.407
	F n, %	61	52.1%	69	57.5%	
Age		52.00	9.54	53.44	10.55	0.278
Match scores						
Regular visits		.80	.53	.79	.56	0.887
Family interventions		.38	.81	.44	.81	0.554
Consulting parents		.70	.59	.74	.60	0.654
Management		.09	.69	.10	.68	0.877
Health promotion		.08	.66	.06	.54	0.806
Administration		−.44	.76	−.44	.77	0.983
Professional consultations		.50	.81	.43	.84	0.538
Research		−.08	.66	−.12	.57	0.626
Burnout						
Burnout EE		20.10	11.78	20.98	11.64	0.567
Burnout DEP		5.58	4.47	7.22	5.28	0.011
Burnout		25.68	14.79	28.19	15.47	0.203

The majority (88.3%) of the responding group were board- certified pediatricians, 18.9% were clinic directors and 24.8% held an academic position. Most of the respondents worked in community clinics (75.7%), 6.9% in hospitals and 17.3% in both. The type of employment contract among the employer and the pediatrician was reported as independent, salaried or a combination of both by 41, 40.2 and 18.6% of respondents respectively.

Burnout was reported by 14 (5.9%), 34 (14.3%) and 30 (12.6%) respondents for DEP only, EE only and both, respectively. Any degree of burnout was reported by 78 respondents, a prevalence of 32.8% (95% CI:26.9–39.1).

Workload and satisfaction from professional activities (both in descending order) and the match between them are presented in Table 2. Professional consultations, regular visits and consulting parents regarding development and inoculations were the three activities with the highest satisfaction and high match scores. Administration and paperwork was the activity with the lowest satisfaction and lowest match score. Tutoring students and conducting research were activities also characterized by low match scores but from the opposite direction: high satisfaction but low workload.

**Table 2:** Workload, satisfaction (in descending order) from professional

	Job satisfaction		Workload		Match score	
	Mean	SD	Mean	SD	Mean	SD
Professional consultations	**3.58**	0.72	3.01	0.79	**0.46**	0.82
Regular visits	**3.52**	0.66	**3.83**	0.56	**0.80**	0.54
Consulting parents: development & vaccinations	**3.47**	0.79	**3.55**	0.70	**0.72**	0.59
Family interventions	3.11	0.86	3.00	0.83	0.41	0.80
Tutoring students and interns	2.88	1.21	2.08	1.07	-0.35	0.48
Community health promotion	2.59	1.05	2.32	0.94	0.07	0.60
Research	2.42	1.17	1.71	0.90	-0.10	0.61
Management	2.27	1.06	2.40	1.08	0.09	0.68
Administration and paperwork	1.95	0.77	**3.22**	0.88	-0.44	0.76
Mean	2.87	0.92	2.79	0.86	0.18	0.66

The three highest scores are bolded

A significant negative correlation was found between task satisfaction and burnout (*r* = − 0.21, *p* < 0.01). Lower burnout was associated with a higher task satisfaction. Similarly, a negative correlation was found between match score and burnout (*r* = − 0.177, *p* < 0.01). The higher the discrepancy between workload and task satisfaction, the higher was the burnout level. Gender was not correlated significantly with any variable.

Age was negatively correlated with burnout (*t* = − 0.202, *p* < 0.01); older pediatricians report less burnout. The number of working hours and ride time to work were positively correlated with burnout (*r* = 0.257, *p* < 0.01, and *r* = 0.153, *p* < 0.05 respectively).

We further investigated differences in workload, task satisfaction, match scores and burnout, in terms of professional status (board-certified and general pediatricians) and managerial status (clinic directors and non-directors). T-tests of independent means were used. Pediatricians who were not board- certified reported a lower satisfaction scores (*t* = − 2.02, *p* < 0.05) and a higher burnout scores (*t* = 2.71, *p* < 0.01) than board- certified pediatricians. Pediatricians who were not clinic directors reported a lower match than clinic directors. (*t* = − 2.842, *P* < 0.001). Work setting (hospital, community, both) was not correlated with satisfaction, match or burnout scores.

We could not find any statistically significant differences (by F tests) between the effects of the different work settings- hospital, community, both - on satisfaction, burnout or match. Salaried pediatricians experienced a higher burnout than those working both as salaried and independent. (F = 5.603, *p* < 0.01), while Independent pediatricians experienced the lowest degree of burnout. Academic status was positively correlated with satisfaction (F = 15.375, *p* < 0.05).

**Fig. 1:**
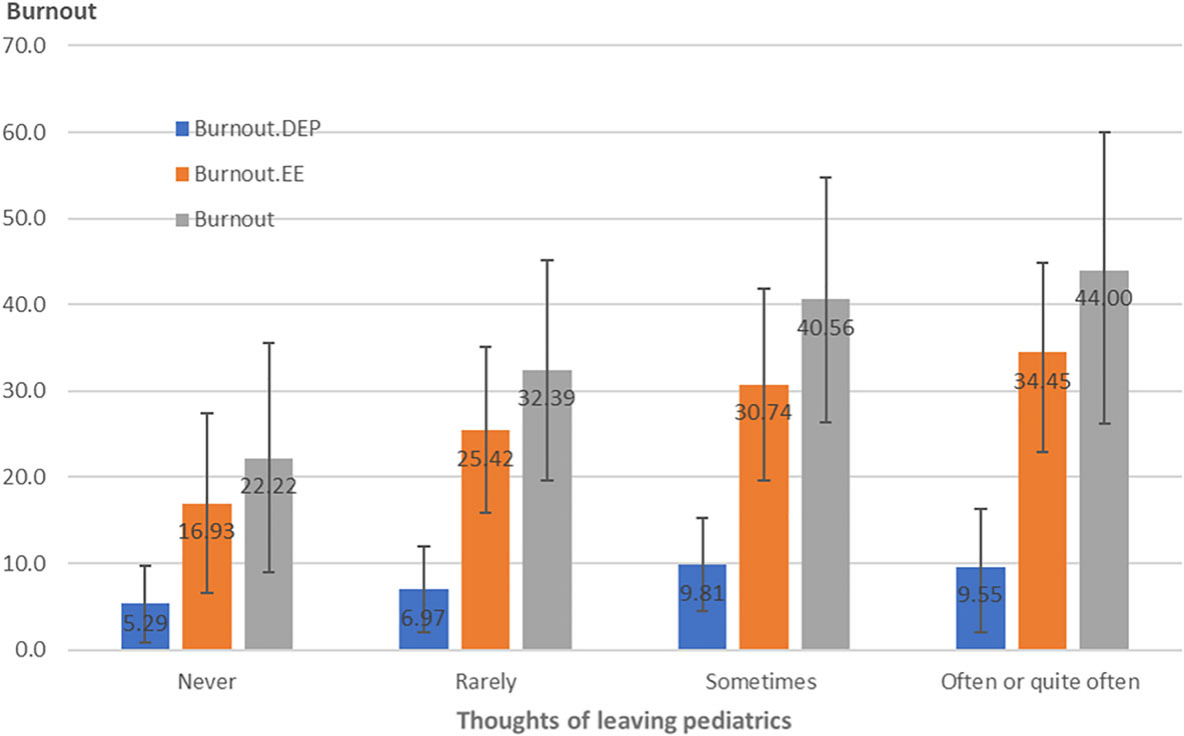
Correlations between quitting pediatrics and burnout

**Fig. 2:**
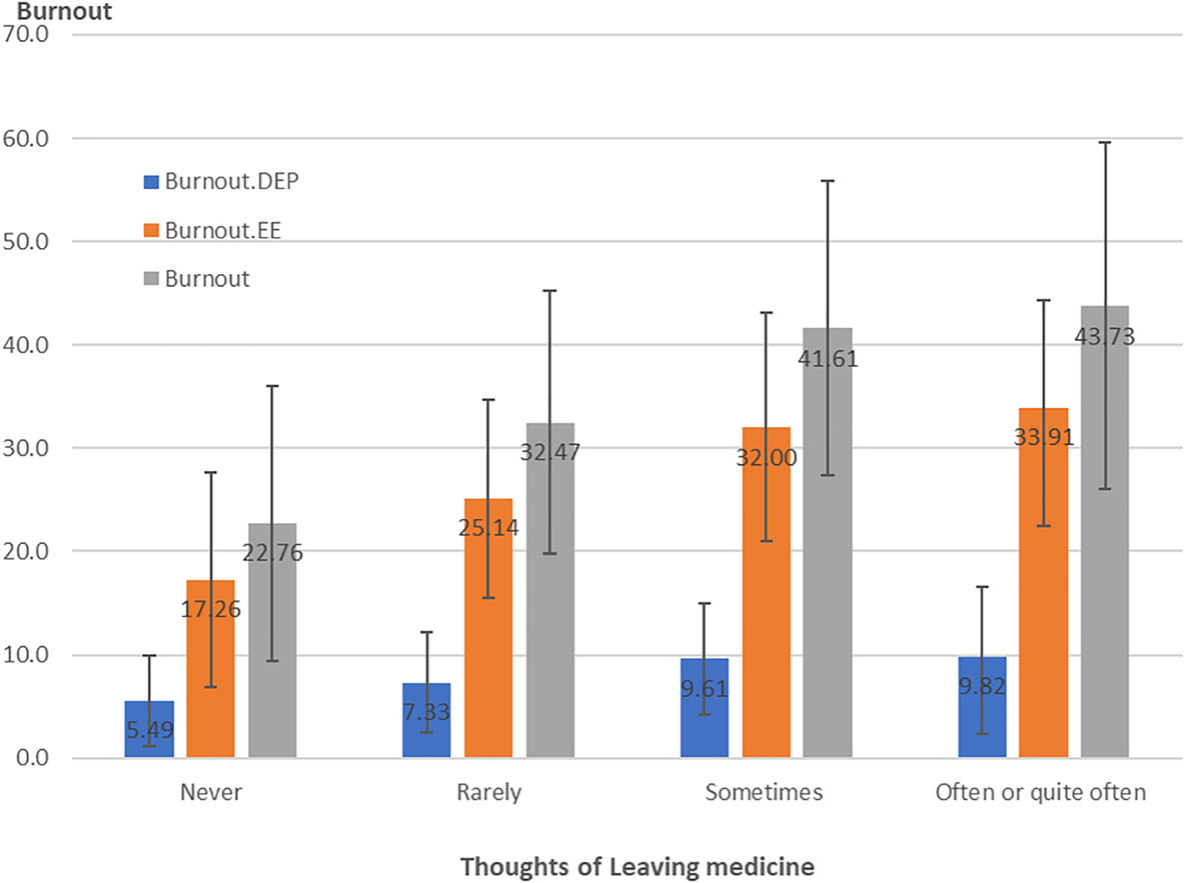
Correlation between thoughts of quitting medicine and burnout

The correlations between burnout and thoughts of quitting the pediatric practice are shown in Fig. 1. Higher burnout EE, burnout DEP and any burnout were associated with more frequent thoughts of quitting pediatrics. The same correlations existed between burnout and thoughts of quitting medicine in general (Fig. 2). Higher burnout was associated with more frequent thoughts of quitting medicine.

The results of the comparison of match scores in our study and those published in 2006 are presented in Table 3. The match scores of the following activities decreased significantly compared with those of 2006: management, professional consultations, community health promotion, tutoring students and research. In all of these activities the discrepancy between satisfaction and workload increased in the time window of 2006 to 2017.

**Table 3:** Comparison of current match scores with those published in 2006

Office activity	“Match” (2016)			“Match” (2006)				
	M	SD	SE	M	SD	SE	difference	*P*
Consulting parents: development & inoculations	0.72	0.59	0.038	0.69	0.68	0.061	0.03	0.155
Family interventions	0.41	0.80	0.052	0.39	0.6	0.053	0.02	0.138
Administration and paperwork	−0.44	0.76	0.050	−0.4	0.76	0.068	−0.04	0.633
Regular visits	0.80	0.54	0.035	0.9	0.4	0.036	−0.10	0.111
Management	0.09	0.68	0.044	0.23	0.51	0.045	−0.14	**0.043**
Professional consultations	0.46	0.82	0.053	0.63	0.54	0.048	−0.17	**0.0074**
Community health promotion	0.07	0.60	0.039	0.29	0.52	0.046	−0.22	**0.006**
Research	−0.10	0.61	0.040	0.23	0.42	0.037	−0.33	**< 0.001**
Tutoring students and interns	−0.35	0.48	0.031	0.3	0.48	0.043	−0.65	**< 0.001**
Mean	0.18	0.66	0.042	0.32	0.23	0.020	−0.14	**0.022**

Significant differences are bolded

## Discussion

Our study documented that almost a third (32.8%) of the pediatricians in this survey experienced high burnout levels. The study also elucidated variables, some of which are new, that are associated with burnout. There is a wide variation in the reported prevalence of burnout among general pediatricians in various locations, ranging between 18 and 46% [16–18]. The higher estimate of 46% was reported in 2015 in the US [17], where there has been a sharp rise from a previous report of 35% in 2012 [19]. In Israel, rates of burnout appear to be somewhat lower than that in the US. For example, reported burnout rate among family physicians in 2015 in the US was 63% [17], while in 2014 in Israel it was 56% [20]. This difference may reflect variable work life balances in Israel and in the US, that can also explain our relatively low burnout rate among pediatricians. In addition, a host of methodological reasons might influence differences in burnout rates in different studies and countries. First, the definition of burnout is based on the selective use of subscales [21]. Some studies use a single dimension of burnout while others use two or three of the MBI sub-scales. Second it has been suggested recently that burnout may be over-diagnosed due to the reliance on studies with extremely low response rates (e.g. 19 and 26% in ref. [17] and [19] respectively). Third, it is plausible that physicians motivated to respond to surveys on burnout are more likely to report burnout [21]Burnout prevalence rates among general pediatricians are lower than in other specialties [19], and lower than that among pediatric subspecialists or intensivists [18, 22]. A possible explanation for the relatively low prevalence of burnout among general pediatricians might be their higher satisfaction with work- life balance [17] compared with other specialties and to other pediatric subspecialties.

The findings of the present study strengthen those obtained in the 2006 study [9], that examined job structure match and burnout, indicating that burnout is associated with the existence of a substantial discrepancy between the level of engagement demanded by the job, and the extent to which pediatricians find satisfaction in what they are doing. Both studies demonstrated that professional consultations have a high match score between satisfaction and engagement, and a high score was inversely related to burnout. The impact of engagement in continuing medical education (CME) was reported in a study among general practitioners in Denmark, where those who were not members of a CME group had a higher likelihood of suffering from burnout [23]. The Danish model for CME includes also Balint groups that have the potential to prevent burnout. However, previous reports on the effectiveness of Balint yielded mixed results, and a systematic review concluded that no evidence was found for the efficacy of Balint groups [24]. In Canada, CME participation of family physicians was recognized as a strategy to reduce stress on the job and was associated with lower levels of burnout [25]. In Israel, participation of pediatricians in CME activities was found to be negatively associated with burnout levels [26].

In addition, the results of the present study support the findings in the study done in 2006 [9], namely that administration and paperwork is the activity with the lowest satisfaction and lowest match score (highest discrepancy) between engagement and satisfaction. Tutoring students and conducting research are two activities also characterized by low match scores but from the opposite direction: high satisfaction but low engagement. Academic status was also correlated with high satisfaction of the pediatrician.

Administrative work has been documented as a negative job characteristic associated with burnout among physicians in Israel and in Canada [25, 27]. On the other hand, the association between physicians’ academic or educational activities and burnout is controversial. Lack of engagement of family physicians in Croatia in education and academic work predicted low personal accomplishment score in the Maslach Inventory [28]. In a similar way, intellectual stimulation predicted higher job satisfaction among Dutch medical specialists [29]. However, teaching and academic activity among family physicians in the Negev area in Israel was not associated with satisfaction [30]. In our study, from the point of view of physicians trained to heal sick people, administrative overload may seem a redundant burden, which is performed at the expense of more important medical tasks. Ideally, such tasks should be reduced to the minimum necessary level to prevent stress and burnout. This can be achieved, where possible, by delegating them to other personnel in the clinic. On the other side, educational or academic activities can provide an opportunity to build one’s professional self-esteem, which may explain the higher job satisfaction demonstrated in our study.

Professional and managerial status in our study were associated with lower burnout and higher match between satisfaction and engagement. In the 2006 study, professional and managerial status were associated with higher satisfaction [9]. In another study evaluating positive and negative work characteristics, board- certified pediatricians reported a higher level of autonomy and clinic directors reported a higher levels of positive work features [27]. These findings suggest that pediatricians having professional and managerial status in the present sample may also have had higher levels of job variety than the other pediatricians, requiring varied tasks and skills (e.g. research, tutoring, community work); and they also performed more duties that were not directly associated with treating and curing patients. It is possible that these added duties, requiring more complex and potentially more challenging types of professional skills [9] were accompanied by a higher feeling of satisfaction.

Younger age was associated with a higher likelihood for burnout. This finding is in accordance with most of the reports in the literature [15, 31]. Older physicians benefit from rich experience and high positions which ensure them respect, reward and less time commitment. Young physicians often have unrealistic expectations of the workload and salary. Therefore, burnout appears to be more of a risk earlier in a physician’s career.

Salaried pediatricians reported the highest level of burnout while those independent reported the lowest. According to our knowledge this is the first report of such a finding, which may be explained by the average higher income of the independent pediatricians, creating a “buffering” effect: at higher levels of work-related satisfaction for current income, the negative effect of emotional exhaustion might be weaker [32].

A positive correlation was documented in our study between burnout and thoughts of quitting pediatrics and medicine in general, supporting recent results among Israeli hospital physicians [33]. The effect of work life balance and job satisfaction on early retirement plans has been reported among US oncologists [34], and an intent to leave the profession was quite prevalent across French physicians and even more so among physicians in emergency medicine [35]. According to our knowledge this is the first documentation of such turnover intentions and its relation to burnout among Israeli pediatricians. Turnover intentions can have a detrimental effect on the pediatric workforce, which is already jeopardized be severe shortage [11].

Our study documented that the levels of work structure match in pediatrics in Israel worsened in the last decade. This is indicated by the significant reductions in the match scores of some of the professional activities between 2006 and 2017, namely management, professional consultations, tutoring students and research. Since lower match scores in our study have generally been shown to correlate with a higher burnout, it can be hypothesized that the level of burnout has also changed across these years. A study performed among Israeli primary care physicians demonstrated the higher burnout levels in 2001 than in mid 1990s [36]. In the US, burnout among pediatricians increased from 35% in 2012 to 46% in 2015: Conversely, satisfaction with work life balance decreased from 62 to 48% in these years [17]. The documented trend in our study, coupled with intentions to leave the practice, is alarming and demands immediate intervention.

Our study has several limitations. First is that the study focused on a sample of Israeli pediatricians, and due to the incomplete response rate (46%) the sample may not be representative of Israeli pediatricians and the findings might be therefore biased and should be interpreted with caution. Thus, replicating the study in different settings is recommended. To partly overcome this limitation, we compared the major characteristics of the study participants with those of Israeli pediatricians as reported by the Israel Ministry of Health [11] and confirmed no significant differences. The second limitation is that the measure of job structure match included subjective self-reported perceptions of workload. Perceived activity level may not reflect actual engagement; therefore, further studies are warranted in which job structure match is assessed by more objective means, such as observations or activity diary. The third limitation is that the burnout scale used in the 2006 study slightly differed from the MBI used in our study. This difference precluded a direct comparison of burnout prevalence. However, we could compare match scores between the time periods, demonstrating a worrying trend.

## Conclusions

The results of the present study highlight some potential intervention measures that may prevent or at least reduce burnout. The pediatrician’s job should be structured, as far as possible, so as to minimize engagement/satisfaction discrepancy and increase the variety and challenge at work. The amount of satisfying, varied and challenging professional duties (such as teaching medical students in the community and participating in research) should be increased. If possible, disliked duties (such as extra administrative work) should be reduced by being delegated to other personnel.

A key to implementation of our study’s findings is to address the physicians and encourage them to diversify their job structure match. This can be done in conferences and by the association’s publications, highlighting the advantages of engagement in management, research and teaching activities, as adjunct activities to the clinical service. Even more important, all four HMOs should be addressed and called upon delegate administrative duties to other personnel and to enable and promote their employed pediatricians in the direction of job diversification.

## Abbreviations

CME: Continuous medical education
DEP: Depersonalization
EE: Emotional Exhaustion
IPRONET: Israel Pediatric Research in Office Setting Network
RR: Response rate
